# Culture Shift: Building an Awareness of Our Mortality

**DOI:** 10.7759/cureus.1969

**Published:** 2017-12-20

**Authors:** Patrick Macmillan, Stephen A Geraci

**Affiliations:** 1 University of California, San Francisco – Fresno Department of Family & Community Medicine, University of California, San Francisco – Fresno Department of Family & Community Medicine; 2 Internal Medicine and Medical Education, East Tennessee State University

**Keywords:** end-of-life care, mortality, immortality, health belief, ethics, palliative medicine, moral perspectives

## Abstract

The end of life discussions can often be difficult for a multitude of reasons. Our culture is pervasive with ideas of mortality, and that medicine can avert this human constant. Medicine, as a field, must embrace our limitations so that we can engage in honest discussions with the families and the patients regarding the end of life care.

## Editorial

I recently met a middle-aged male in the hospital who was recently diagnosed with widely metastatic adenocarcinoma of the lung, a terminal condition, however, with the advances of the immunotherapy and other targeted approaches, life can possibly be extended with hopes of the quality of life. When I spoke to him, he understood that he had lung cancer, but was confident he could 'beat it'. He brimmed with hope and relayed to me that he would be undergoing the chemotherapy soon. I addressed the seriousness of his condition, but he again assured me that he would 'beat' cancer. He had a bright and wide smile on his face, making it difficult to have a forthright conversation with him in spite of my best efforts. However, I thought he had the right to maintain a maximal degree of hope that he would overcome this malady, despite my certainty, that the disease would eventually claim his life. I closed in typical fashion by reassuring him that we would support him through his treatment on an outpatient basis and bid him farewell.

We saw him sometime later when he was again admitted to the hospital. He had received the chemotherapy and was now gravely ill and septic. Vanished from his face was any smile or enthusiasm. As he lay there with tubes feverishly pumping life-saving antibiotics in his veins, I wondered what he was thinking and feeling. The good news, there was some degree of the tumor slowing but the patient looked very sick. His voice struggled to produce even a whisper. He had lost more weight and was unable to ambulate. He remembered seeing me so the conversation naturally flowed as we discussed his goals. Although his spirit seemed on life support, his resolve to 'beat' cancer continued inexorably. He was looking forward to more chemotherapy and expected it would be soon. As I explained his condition, being septic and having no immune reserves to fight off the most benign microscopic invaders, his pupils began to enlarge, a sign that his sympathetic nervous system is activated from excitement or fear. For the first time, I began to see some reality of his diagnosis and prognosis permeate into his conscious mind. I asked him if we could meet with his family to discuss his goals of care and he agreed. Frankly, I had to be very blunt with him about the prognosis, given his compromised functional status.

The patient remained in the hospital for over a month but was eventually discharged home with his sister in hospice care.

As a student of history, I always search for the references to position popular culture into its proper historical context. Most of us can recount our childhood history lessons and tales of the 16th-century quest of the Spanish explorer Ponce De Leon to find the 'Fountain of Youth' in North America. The Greek historian Herodotus, the 'Father of History', wrote about such a fountain as early as the 5th-century B.C. Most major religions have a tenet of an 'eternal soul', reincarnations or other mechanisms that provide their follower's immortality in some form. Today, the media continuously bombard us with diets, exercise programs, supplements and even prescription medications, claiming that each will prolong our lives. Their popularity attests to the commonly misguided concept that immortality should be possible and constitutes a laudable goal.

The human psyche is largely woven into the fabric of immortality and everlasting youth. We all sense connections to our bodies, minds and physical surroundings as we traverse the myriad paths of life. Survival is a primal instinct. Yet, when facing the inarguable reality of the physical mortality, most people acknowledge, usually with the significant emotional detachment that their physical body, as they know it, has distinct limits. A seemingly unresolvable conflict that few people choose to contemplate until forced to do so. There are many examples of such contradictions in nature; a dichotomy that stretches our ability to see the world in stark or black and white terms; a virtual paradox that interferes with the circuitry that allows us to interpret the world in simple schemes. The sun, with its seemingly infinite power, provides life-giving energy and moves our planet, but excessive exposure can also damage our deoxyribonucleic acid (DNA) causing life-threatening cancers in predisposed individuals. The food is essential for life, but too much food, resulting in obesity, can lead to diabetes and fatal heart disease. Mortality and immortality, by all accounts, work to oppose one another. But do they?. Can our fear or refusal to accept mortality create irrational expectations on the medical profession?. Does our medical training create a blind spot?. Is extending the life or preserving immortality the only measure of success?. Somewhere a balance needs to be negotiated between the two concepts.

My profound interest in the mortality dichotomy was sparked after reading Atul Gawande’s book, 'Being Mortal' [1]. I was struck by his way of looking at mortality and its natural connection to our human experience. Gawande carefully unwinds the experience from Tolstoy’s timeless novel, The Death of Ivan Ilyich, and illustrates how our medical training might create a dubious notion that Ilyich’s condition would be easily remedied through 21st-century medicine. Missing from the encounter is the idea that there are limits to what we can do as physicians to alter the course of certain physical diseases. Every day, I am struck by the perception of the patients, families, and even some medical professionals that medicine has the curative key to any physical affliction. This misperception stems from a combination of sources, including the media, religious orders, and the cultural expectations of excellence and perfection from those who serve us.

Occasionally it does appear that modern medicine has cracked the code of mortality. There are many advances in cancer care, life-extending medications and, interventions that allow individuals to live today when only fifty years ago, it surely would have meant imminent fatality. Additional examples include the human immunodeficiency virus (HIV) infection and the 'miracle' of anti-retroviral therapy or left ventricular assist devices in the setting of advanced heart failure. Deepak Chopra, the American author, and champion of 'integrative and alternative medicine' wrote the book, 'Ageless Body, Timeless Mind' [2]. In this book, he writes about defying the aging process on a cellular level.

Our human experience bathes in the pool of immortality, whether it exists on the physical or metaphysical plane. I see hospital patients every day who are facing their own mortality and asking the question, 'How much time do I have left?'. They are facing a defiant riddle that medical science cannot solve. Many times the questions are less dramatic and involve probing the disease course, the prognosis and options slow down the progression and lessen the physical and emotional burden. It’s often a delicate conversation, but that is the one I take very seriously.

A recent abstract by Keng, et al. [3] demonstrated a higher mortality rate in the individual cancer patients requiring prolonged mechanical ventilation compared to those who do not. The authors concluded that “cancer status and weaning outcome were the most important determinants associated with long-term mortality in the cancer patients requiring prolonged mechanical ventilation.” The authors also suggested that palliative care is consulted on such patients. The medical effectiveness of drugs is always defined by their effect on mortality and morbidity. Should the quality of life be part of the equation?. I often construct my goals of care discussions in a quality versus quantity proposition. Generally speaking, the patients opt for the quality. Is living longer the goal or would the idea of living better be preferable?.

There is an element of the culture shift that the field of medicine faces. It involves the 'taboo talk' about death and dying, about mortality. I have spoken to many individuals throughout my career about the health, illness, and death. In all instances, I have found that honesty is always appreciated. I am thrilled when we have the treatment options for our patients. However, I am equally comforted knowing that the options exist that offer hope and foster the quality of life when the road of curative measures narrows. Telling a patient 'we have no more options' is never acceptable because we can always do something to alleviate the suffering of an individual, even when extending the life is no longer possible.

We presently have no protocol or pathway to follow regarding how we negotiate our own subjective feelings of immortality. How then can we guard against giving false hope in the face of so-called medical futility?. It must be our humanity, our compassion, and our divinity. Striking a balance between everlasting life on earth and the physical limits is essential to our profession. Is prolonging life the goal or is fostering a patient’s quality of life the higher outcome?. A patient’s death should not be seen as a failure but as an inevitability. 

We are standing on the shoulders of the history. Our culture constantly changes and adjusts to the technology and the shifts in time. The mythology, dreams, and spirituality shower us with the reassurance that this life has to mean, much greater than the superficial barometers we often use to measure success. Embracing mortality allows us to be at peace with ourselves, and our world, along with whatever awaits us beyond our own dimensions.
